# Effectiveness of Botulinum Toxin in the Treatment of Neuropathic Pain: A Literature Review

**DOI:** 10.7759/cureus.46848

**Published:** 2023-10-11

**Authors:** Anushka Dekhne, Harmin D Goklani, Neel Doshi, Rishabh Baskara Salian, Siddharth Kamal Gandhi, Priyansh Patel

**Affiliations:** 1 Department of Internal Medicine, American University of Antigua, Antigua, ATG; 2 Department of Internal Medicine, Ahmedabad Municipal Corporation Medical Education Trust Medical College, Ahmedabad, IND; 3 Department of Internal Medicine, Pravara Rural Medical College, Loni, IND; 4 Department of Internal Medicine, Kasturba Medical College, Mangalore, IND; 5 Department of Internal Medicine, M.P. Shah Government Medical College, Jamnagar, IND; 6 Department of Internal Medicine, Medical College Baroda, Vadodara, IND

**Keywords:** complementary & alternative medicine, pain management, neuralgia, botox injections, botulinum toxins

## Abstract

Neuralgia is characterized by chronic pain resulting from damage or diseases in the somatosensory system, including nerves responsible for transmitting sensory information. Current treatments for neuropathic pain, which is a type of neuralgia, have limited success rates and can cause unwanted side effects. Since 1989, botulinum toxin-A (BTX-A), derived from the potent neurotoxin *Clostridium botulinum*, has been used to treat neuropathic pain in humans. BTX-A has shown analgesic effects by inhibiting the release of neurotransmitters involved in pain transmission. This review aims to evaluate the effectiveness of BTX-A in various types of neuralgia. The research question guiding this review is whether BTX-A is safe and effective in reducing pain in different types of neuralgias. To conduct this review, a literature search was performed using the PubMed, Medline, and PubMed Central databases. The search strategy included relevant keywords related to BTX-A, neuralgia, and neuropathic pain. After screening titles, abstracts, and full texts, a total of 30 articles were included in the review. These studies examined the efficacy of BTX-A in various conditions such as postherpetic neuralgia (PHN), auriculotemporal neuralgia (ATN), occipital neuralgia (ON), leprosy-induced neuropathic pain (LIN), focal painful neuropathies, complex regional pain syndrome (CRPS), trigeminal neuralgia (TN), and neuropathic pain associated with spinal cord injury. However, further research is needed to enhance our understanding of the optimal use of BTX-A in specific neuralgias. It is important to acknowledge the limitations of the included studies. Nevertheless, BTX-A might be considered a viable treatment option for neuralgia.

## Introduction and background

A lesion or illness affecting the somatosensory system can lead to neuralgia or neuropathic pain. The somatosensory system enables us to perceive touch, pressure, pain, temperature, position, movement, and vibration [1]. It consists of various types of nerves, including thermoreceptors, mechanoreceptors, chemoreceptors, proprioceptors, and nociceptors, which are found in the skin, muscles, joints, and fascia. These nerves transmit sensory information to the spinal cord, which then relays it to the brain for processing. Conditions such as amputation, postherpetic neuralgia (PHN), trigeminal neuralgia (TN) [2], painful radiculopathy, diabetic neuropathy (DN), human immunodeficiency virus (HIV) infection, leprosy-induced neuralgia (LIN) [3], peripheral nerve injury pain, piriformis syndrome [4], auriculotemporal neuralgia (ATN) [5], spasticity, spinal cord injury, and intractable chronic occlusion [6] are commonly associated with neuropathic pain. It is estimated that about 17% of the population experiences neuropathic pain, and among individuals with spinal cord injuries, 75%-81% suffer from pain, with 53% experiencing neuropathy [7]. Chronic pain development and persistence remain significant clinical challenges, and our current understanding of these processes is limited. Chronic pain affects one in five Europeans, leading to lifestyle modifications due to its various causes [8]. The absence of effective treatments further exacerbates the issue. While numerous pharmacological and non-pharmacological therapies have been proposed, their outcomes have been unsatisfactory. Pharmacological options, such as non-steroidal anti-inflammatory drugs (NSAIDs), opioids, antidepressants, and anticonvulsants, have been used, but they can result in unpleasant events and reduced analgesic effectiveness. These medications also carry substantial adverse effects, and success rates of treatment are estimated to be between 30% and 50% [9]. Neuropathic pain profoundly affects patient perceptions, thoughts, emotions, and actions [9]. Clinicians face difficulties in managing neuropathic pain, which limits the benefits of pain reduction, resolution of underlying conditions, and the achievement of a good quality of life through rehabilitation [10]. Botulinum toxin-A (BTX-A), derived from the potent neurotoxin *Clostridium botulinum*, offers a potential treatment option. BTX-A prevents the release of acetylcholine (ACh) from neuromuscular junctions, resulting in muscle relaxation [11]. Studies in animals have shown that BTX-A also inhibits the release of various other neurotransmitters like substance P, calcitonin gene-related peptide, and glutamate, which have effects on presynaptic vesicles in neurons. Importantly, the analgesic activity of BTX-A is distinct from its muscle-relaxing effects [11]. BTX-A has been used since 1989 to treat neuropathic pain in humans and has shown effectiveness in various conditions, including headaches, migraines, arthritic pain, cerebral palsy with acute sialadenitis, PHN, TN, painful radiculopathy, DN, HIV infection, LIN, amputation, peripheral nerve injury pain, piriformis syndrome, ATN, spasticity, spinal cord injury, and intractable chronic occipital neuralgia [11]. The objective of this review is to describe the efficacy of botulinum toxin (Botox) in treating different types of neuralgia. It has demonstrated promise as a potential therapeutic option. However, further research is needed to determine the optimal use of Botox in specific types of neuralgia. Despite its limitations, Botox has shown potential as a suitable treatment option for neuralgia.

Methodology

We hypothesize that Botox is safe and effective in reducing pain in different types of neuropathic pain/neuralgias. The study involved a literature assessment of the effectiveness of Botox in the treatment of neuralgia using the regular and medical subject headings (Mesh) database on PubMed, MEDLINE, and PubMed Central. The search was conducted and a search string of keywords related to botulinum toxin, neuralgia, and neuropathic pain were used. Studies not in English and studies published before 2008, were excluded. Further, non-observational studies, meta-analyses, and systematic reviews were not included in this review. Titles and abstracts were screened to check for duplicates and to determine their relevance to the research question. The screened articles were then passed through a full-text screening that was performed to check for eligibility, relevance, and outcomes. The data were analyzed to identify common findings and trends across the studies. A total of 30 articles were included from the literature search.

## Review

Pathophysiology

**Figure 1:**
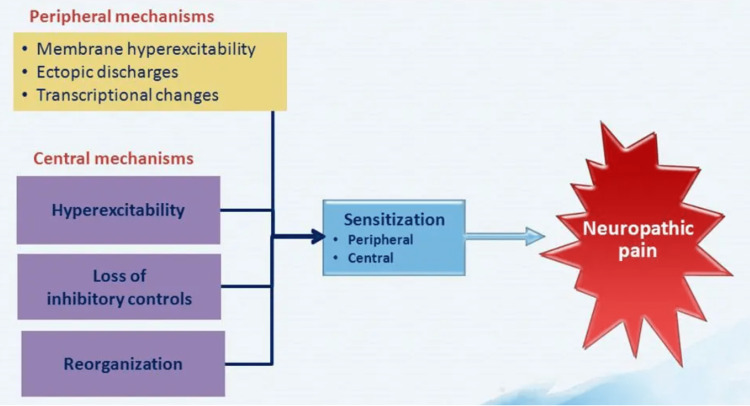
Pathophysiology of neuropathic pain Image credits: Anushka Dekhne, Priyansh Patel

Changes in pain signaling and sensory processing at different levels of the nerve system lead to neuralgia. An imbalance between excitatory and inhibitory signaling results from the alteration of the electrical characteristics of sensory neurons by peripheral neuropathy. Chronic neuropathic pain is characterized by alterations in pain signaling that occur as a result of imbalances and changes in the transmission of sensory signals by dorsal horn neurons in the spinal cord. The dorsal horn neurons play a crucial role in processing and transmitting sensory information from the periphery to the brain [12]. Sodium, calcium, and potassium channels, as well as altered ion channel activity and expression, are involved in neuropathic pain. The spinal cord's increased sodium channel expression and functionality increases excitability and the release of neurotransmitters and results in peripheral sensitization. Continuous pain and hypersensitivity result from the loss of potassium channels and the creation of ectopic activity in damaged fibers [13]. Increased excitability and enlarged receptive fields as a consequence of modifications to second-order nociceptive neurons cause central sensitization as shown in Figure 1. Inhibitory interneurons are dysfunctional, which has an impact on inhibitory modulation as well. Affected sensory processing and aberrant pain perception come from the disruption of the equilibrium between inhibitory and excitatory signaling [14].

Current therapy

Pharmacologic therapy is the first-line treatment option for patients suffering from neuralgia [15]. The anticonvulsant medicine carbamazepine is the most often prescribed drug [15]. However, carbamazepine's efficacy wanes with time in certain people [15]. For patients with TN who do not react to or are unable to take carbamazepine, oxcarbazepine (anti-epileptics), and baclofen (a muscle relaxant) are being utilized more often as first-line treatment [16]. Other drugs include phenytoin, gabapentin, pregabalin, clonazepam, valproic acid, and lamotrigine (anti-epileptics). In Tennessee, pain treatment options include the sodium (voltage 1.7) blockers vixotrigine and eslicarbazepine (an active metabolite of oxcarbazepine) [16].

Complications

Neuropathic pain or neuralgia can have various complications, including the following.

Sensory Abnormalities

People with neuropathic pain may feel altered sensations such as numbness, tingling, or hypersensitivity to touch. These sensory impairments can impair balance, coordination, and movement, raising the risk of slips, falls, and other accidents [17].

Lower Quality of Life

Constant pain and its symptoms can greatly lower a person's quality of life. It might result in fewer social connections, a withdrawal from interests and activities, and an overall loss of physical and mental health [17].

Emotional and Psychological Effects

Having chronic pain can lead to emotional problems such as worry, melancholy, frustration, impatience, and mood changes. The psychological effects might aggravate how painful an event feels and make it harder to successfully manage the disease [17].

Sleep Disturbances

Neuropathic pain can significantly impact sleep patterns, causing disruptions in falling asleep, staying asleep, and obtaining restful sleep. Sleep problems associated with neuropathic pain can exacerbate discomfort, impair daily functioning, and contribute to the development of additional health issues [17].

Functional Restrictions

Severe neuropathic pain can impede physical function and mobility, making it difficult to carry out daily tasks, work, or participate in leisure activities. The limitations can lead to decreased independence and a loss of productivity [17].

Side Effects of Medications

It can cause drowsiness, constipation, dizziness, nausea, and reliance [17].

BTX-A as a treatment option

BTX-A, which is derived from strains of *Clostridium* *botulinum*, acts by inhibiting the release of ACh, substance P, calcitonin gene-related peptide, and glutamate at neuromuscular junctions, resulting in muscle relaxation. However, its effects extend beyond muscle relaxation. These neurotransmitters play a role in pain signaling and affect the presynaptic vesicles of neurons [1]. The analgesic action of BTX-A is distinct from its muscle-relaxing properties. Research has shown that BTX-A can alleviate pain independently of its effects on muscle relaxation. It has been used effectively in clinical practice for the treatment of various conditions [1]. These include PHN, TN, painful radiculopathy, DN, HIV infection, LIN, amputation, peripheral nerve injury pain, piriformis syndrome, ATN, spasticity, spinal cord injury, and intractable chronic occipital neuralgia. The successful use of BTX-A in these conditions demonstrates its potential as a treatment option beyond its originally recognized applications [1].

To evaluate the effectiveness of Botox in pain management, our review examined multiple studies investigating its use in the treatment of various neuropathies and neuralgias. Among the included studies, two specifically focused on the efficacy of Botox in relieving pain in patients with PHN. One of these studies compared the use of Botox with single nerve root pulsed radiofrequency therapy (RFT) for treating PHN [11,18,19]. The study assessed the effectiveness and safety of each approach. The results revealed that both treatment groups experienced similar levels of pain reduction. However, it was noted that RFT posed challenges in terms of administration and cost compared to Botox. These findings aligned with our initial hypothesis, confirming that Botox is a safe and effective pain reliever for neuralgia. These findings contribute to the growing body of evidence supporting the use of Botox as a viable treatment option for alleviating pain in PHN and potentially other neuralgic conditions [11,18,19]. The second research evaluated the efficacy of Botox in the treatment of pain in patients with peripheral DN and PHN, and it also validated our hypothesis by demonstrating that BTX-A is beneficial in lowering pain in both diseases [20]. These studies showed that BTX-A is effective in treating TN [21,22]. Another study described the usage of various dosages of BTX-A in TN patients, contrasting it with TN patients receiving placebo medication. It highlighted how, starting in week 1, and continuing through week 8, the 25 units (U) and 75 U groups' pain visual analog scale (VAS) ratings were considerably lower than those of the placebo group. Between the 25 U and 75 U groups, there was no discernible change in VAS throughout the research. Thus, it said, low dosages (25 U) and large doses (75 U) had comparable short-term effectiveness [23-25].

In a study conducted by Tereshko et al., the use of BTX-A in patients with ATN was examined. These patients received therapy involving the administration of BTX-A. The effects of BTX-A were observed to manifest approximately seven to 10 days after the injections [5]. Among the participants, three individuals experienced complete pain relief that lasted for up to six months, four individuals had partial pain reduction lasting for three months, and two individuals showed a poor response with a shorter duration of pain relief (30-45 days) [5]. Various pain scale scores were assessed, and the overall results demonstrated significant improvement one month after the treatment. Patients reported less pain interference with daily activities, mood, job performance, relationships, sleep, pleasure of life, and facial contact. Based on the findings of the study, it was concluded that BTX-A is a safe and effective therapy for ATN [5]. A study conducted by Kim et al. investigated the use of BTX-A in patients with unilateral chronic ON. The participants received injections of BTX-A at the sites of pain. The study confirmed our hypothesis by demonstrating that BTX-A was both safe and effective in the treatment of ON [6].

Injecting BTX-A has been shown to be effective in treating resistant leprosy patients with persistent neuropathic pain, according to research by Sousa et al. With immediate pain alleviation in the first week and stabilization after that, the VAS indicated a considerable decrease in pain intensity from day 10 to day 60 [3]. In leprosy patients who acquired neuropathic pain, the research suggested using botulinum toxin therapeutically. The only noticeable adverse effect of the toxin, particularly when given to the afflicted nerve regions and extremities, was temporary mild to severe discomfort during injections in many individuals, indicating that the toxin had excellent tolerance. The data support our hypothesis by indicating that BTX-A is a viable treatment choice for leprosy patients with persistent neuropathic pain [3].

In a trial conducted by Ranoux et al., patients with localized painful neuropathies and mechanical allodynia were randomly assigned to either the BTX-A or placebo groups. Both groups received injections with comparable numbers of injection sites and overall volumes [26]. The results of the trial demonstrated that BTX-A had a significant impact on reducing the average level of pain compared to placebo. The pain relief effects of BTX-A became apparent from the second week and persisted for up to 14 weeks. Moreover, a higher response rate and some patients even reported complete pain alleviation with BTX-A treatment [26]. Additionally, BTX-A demonstrated the ability to decrease allodynia (pain due to non-painful stimuli) and improve the quality of life and various neuropathic symptoms experienced by the patients. These findings provide strong evidence supporting the safety and efficacy of BTX-A in the treatment of localized painful neuropathies [26]. The trial's results align with our initial hypothesis, further confirming that BTX-A is a valuable therapeutic option for managing neuropathic pain in specific localized conditions [26].

In a study conducted by Choi et al., the combined use of levobupivacaine 0.25% and Botox for lumbar sympathetic block (LSB) in patients with CRPS was examined. The research aimed to assess the effectiveness of this treatment approach [27]. The results of the study demonstrated significant reductions in pain intensity and the Leeds assessment of neuropathic symptoms and signs (LANSS) score at the two-month follow-up after LSB with Botox [27]. Additionally, improvements were observed in skin color, disappearance of allodynia, and alleviation of coldness. The study also suggested that Botox could create an effective and long-lasting sympathetic blocking effect in individuals with CRPS [27]. Patients with spinal cord injury-related neuropathic pain were included in a trial by Han et al. and either the BTX-A or placebo group received injections of saline into the painful location. In comparison to the placebo group, the BTX-A group demonstrated statistically significant VAS score decrement at four and eight weeks after the injection [28-30]. A summary of the current literature is listed in Table 1.

**Table 1:** Summary of the current literature BTX-A: botulinum toxin type A, CT: computed tomography, ATN: auriculotemporal neuralgia, ON: occipital neuralgia, TN: trigeminal neuralgia, RCT: randomized control trials, RF: radiofrequency, PHN: post-herpetic neuralgia, CRPS: chronic regional pain syndrome, SCI: spinal cord injury

Studies	Comments
Intiso et al. [1]	Using BTX-A in neurorehabilitation can effectively treat neuropathic pain. The authors delve deeper into presenting an overview of the evidence that supports BTX-A use in this context, along with its risks and benefits. As a result, this article proves to be a resource for clinicians contemplating using BTX-A to alleviate pain in their patients.
Capon et al. [2]	The study effectively suggests that the BTX-A injections may have worked by being transported retrogradely to the trigeminal ganglion, where they blocked the release of pain-signaling neurotransmitters. As a result, it can be concluded that BTX-A may be an effective treatment for refractory post-traumatic trigeminal neuropathy.
De Sousa et al. [3]	This study investigated the use of BTX-A in treating chronic neuropathic pain in patients with refractory leprosy. The conclusion I derived from the study was that BTX-A effectively reduced pain in patients with chronic neuropathic pain due to leprosy. Additionally, it effectively relieved pain with improved quality of life for these patients.
Yan et al. [4]	This research is quite effective in assessing the impact of CT-guided injections, both with and without BTX-A, for treating piriformis syndrome. The findings suggest that CT-guided injections combined with toxin tend to result in a positive response and longer-lasting effects compared to patients who only receive CT-guided injections without botulinum toxin. This study is significant as it supports the use of BTX-A as a treatment option for piriformis syndrome.
Tereshko et al. [5]	The study evaluated the effectiveness of BTX-A in treating ATN. Provides valuable evidence to support using BTX-A as a treatment for ATN. BTX-A may be a preferred treatment option for ATN patients who are not candidates for surgery or have not had success with other treatments.
Kim et al. [6]	Despite the research being conducted by a small group, the results are significant and can make a difference in the medical field. This study is one of the few medical studies that evaluate the use of BTX-A for the treatment of chronic ON. To effectively validate the treatment of chronic ON using BTX-A, further studies are needed from a large group.
De Icco et al. [7]	The findings of this study offer proof that BTX-A is successful in decreasing spasticity and alleviating pain among patients. Moreover, BTX-A has shown an impact on the summation threshold, which assesses the sensitivity of spinal cord nociceptive pathways.
Rojewska et al. [8]	It is a well-written review article that discusses the effects of BTX-A on the relationship between glia and neurons under neuropathic pain conditions. This review addresses the effects of BTX-A on the relationship between glia and neurons under neuropathic pain. The authors concluded that BTX-A is an effective and promising treatment option for neuropathic pain. However, to further validate these findings, more research is needed to help comprehend the mechanisms by which BTX-A exerts its analgesic effects.
Leese et al. [9]	In this study, the authors provide a significant study outcome that is useful in treating chronic pain. Their study is exciting because it describes methods that can be used in producing non-paralytic botulinum molecules, which are significant in treating chronic pain. The authors also gave more insights in their study with evidence that non-paralytic BTX-A molecules could be a safe and effective treatment for chronic pain. Healthcare organizations can benefit from this critical information as they explore the best medical methods for treating chronic pain.
Datta Gupta et al. [10]	Neuropathic pain arising from injury or dysfunction is a form of pain that affects 10% of the general population. Recognizing this, the article offers an examination of the evidence pertaining to the application of BTX-A for neuropathic pain management. This research serves as a resource for practitioners and researchers curious about employing BTX-A in treating neuropathic pain. It presents an overview of evidence while also acknowledging the limitations found within existing literature.
Wei et al. [11]	BTX-A has been proven to be effective and safe for treating TN and peripheral neuropathic pain. The research was effective in distinguishing a clear and notable effect of BTX-A, showing that it was superior to a placebo based on pain intensity. The pain intensity was recorded to have a lasting effect of up to three months. While this study was effective, the validity of the results is disputable considering the limited sample size and heterogeneity, making application in a large population limited. However, further and larger well-designed RCTs can help further validate these findings.
Baron et al. [14]	I agree with the author’s viewpoint that personalized medicine holds the key to the future of treating neuropathic pain. By gaining an understanding of the pain mechanisms in each patient, we can create treatments that are more precise and impactful. Achieving this will necessitate a partnership between healthcare professionals and researchers alongside advancements in tools and biomarkers.
Chen et al. [18]	The study was well designed in the evaluation of the effectiveness of BTX-A and pulse RF. The study was accurate and comprehensive, finding that both BTX-A and RF are effective in reducing pain and improving the quality of life for patients with PHN. The study also suggested with the two methods, BTX-A can be more reliable due to its ease of administration and lower cost.
Moon et al. [20]	This article holds importance as it presents evidence supporting the effectiveness of BTX-A in treating chronic challenges like neuropathic pain. The study further explains the success of this treatment among patients previously not responding to treatments. The study concludes with a convincing proposition that incorporating BTX-A into the range of treatment options for neuropathic pain could be highly beneficial.
Ranoux et al. [26]	This groundbreaking research offers evidence that BTX-A is a highly effective treatment for chronic neuropathic pain. While BTX-A is already utilized for conditions such as muscle spasticity and migraines, this study indicates its potential value as an additional treatment option, specifically for chronic neuropathic pain.
Choi et al. [27]	Based on the research carried out by the authors of this article, it is clear that BTX shows effectiveness in reducing the intensity of pain and improving symptoms and signs. Additionally, the study suggests that BTX could be an effective treatment for CRPS, with a lasting impact compared to other sympathetic blockers.
Chun et al. [29]	In the study of the treatment of at-level spinal cord injury pain with BTX-A, the authors of this paper did an outstanding job in effectively evaluating the use of BTX-A for the treatment of at-level SCI pain. Their approach was effective and detailed, making this study significant. It provides the subcutaneous injection of BTX-A may be a feasible approach for the control of at-level SCI pain and is worthy of further study.

Limitations

Our evaluation includes case reports and randomized controlled trials, the majority of which made it challenging to pinpoint the precise time at which BTX-A's effects began to manifest and how long they lasted. The study's drawback stems from the study's limited participant population. Only three of the included studies were placebo-controlled. Furthermore, a comparison of various dosages and injection procedures, as well as large-scale, multicenter, long-term follow-up, placebo-controlled studies, are therefore necessary in the future. Before including the use of BTX in clinical scenarios, extensive research is still required for the effectiveness of BTX.

## Conclusions

The utilization of BTX-A in the treatment of various forms of neuralgia and neuropathic pain shows promising outcomes in terms of reducing pain and improving overall patient outcomes. BTX-A's mechanism of action involves more than just its muscle relaxation properties. It functions by inhibiting the release of neurotransmitters, providing pain relief beyond its direct effects on muscle tissue. However, further research is required to determine optimal dosing, treatment protocols, and the long-term effects of BTX-A in the management of neuralgia. Despite the need for additional investigation, BTX-A shows significant potential as a valuable treatment option for various types of neuralgia. Its ability to alleviate pain and improve outcomes in these conditions holds promise for individuals suffering from neuropathic pain.
